# 基于生物信息学探索DKK1在肺腺癌发生中的作用

**DOI:** 10.3779/j.issn.1009-3419.2023.101.22

**Published:** 2023-08-20

**Authors:** Ruijiao LU, Yuxia LI, Abuduhailili XIEYIDAI, Tingting YU, Yangchun FENG

**Affiliations:** ^1^830011 乌鲁木齐，新疆医科大学第三临床医学院（附属肿瘤医院）医学检验中心，; ^1^Medical Laboratory Center,; ^2^830011 乌鲁木齐，新疆医科大学第三临床医学院（附属肿瘤医院）肺内科一病区; ^2^First Department of Lung Cancer, the Third Clinical Medical College (Affiliated Cancer Hospital) of Xinjiang Medical University, Urumqi 830011, China

**Keywords:** 肺肿瘤, DKK1, 预后, Lung neoplasms, DKK1, Prognosis

## Abstract

**背景与目的:**

肺癌是我国最常见的恶性肿瘤，肺腺癌（lung adenocarcinoma, LUAD）是肺癌的主要类型，严重威胁着人民的生命健康，目前关于血清分泌型蛋白1（Dikkopf 1, DKK1）在LUAD中的作用研究较少，本研究旨在通过生物信息学方法探究DKK1在LUAD发生发展中的作用及潜在的预后价值。

**方法:**

应用基因型组织表达（genotype-tissue expression, GTEx）、癌症基因组图谱（The Cancer Genome Atlas, TCGA）数据库和肿瘤与免疫系统交互网站（tumor-immune system interactions database, TISIDB）等多个数据库，对DKK1在LUAD中的表达、临床病理特征、免疫细胞浸润、预后和甲基化等进行分析，同时应用LinkedOmics数据库分析DKK1的共表达基因及其功能富集。收集2016年至2017年于新疆医科大学附属肿瘤医院行手术治疗的59例石蜡包埋的LUAD患者病理样本，通过免疫组织化学试验（immunohistochemistry, IHC）进行表达预后验证。

**结果:**

生信分析结果显示DKK1在LUAD组织的表达水平高于正常组织，晚期癌症中的表达高于早期阶段，实验验证后发现59例LUAD中阴性表达15例（25.4%），弱阳性表达18例（30.5%），强阳性表达26例（44.1%）。DKK1的不同表达情况与甲基化、预后以及多种免疫细胞的活动相关。功能富集显示DKK1可能参与表皮发育、细胞-基质连接等过程，京都基因与基因组百科全书（Kyoto Encyclopedia of Genes and Genomes, KEGG）分析表明DKK1与ABC转运蛋白相关。生物信息学分析及临床病例标本显示DKK1高表达与LUAD患者较差的预后有关。

**结论:**

DKK1在LUAD中高表达，与患者预后不良有关，并且DKK1与肿瘤免疫细胞浸润和通路密切相关。DKK1可能是LUAD潜在的预后标志物和免疫治疗新靶点。

## Abstract (en)

**Background and objective:**

Lung cancer is the most common malignant tumor in China, lung adenocarcinoma (LUAD) is the main type of lung cancer, which is a serious threat to people's life and health. At present, there are fewer studies on the role of Dikkopf1 (DKK1) in lung adenocarcinoma. The aim of this study was to investigate the role and potential prognostic value of DKK1 in the development of lung adenocarcinoma by bioinformatics methods.

**Methods:**

Several databases, such as genotype-tissue expression (GTEx), The Cancer Genome Atlas (TCGA) and tumor-immune system interactions database (TISIDB), were used to analyze the expression, clinicopathological features, immune cell infiltration, prognosis and methylation of DKK1 in lung adenocarcinoma. Then, linked immune cell infiltration Omics database was used to analyze the co-expressed genes of DKK1 and their functional enrichment. Finally, 59 pathological samples of paraffin-embedded lung adenocarcinoma patients who underwent surgery at the Affiliated Cancer Hospital of Xinjiang Medical University between 2016 and 2017 were collected for the validation of the prognostic value of expression by immunohistochemistry (IHC) test.

**Results:**

The results of bioconfidence analysis showed that the expression level of DKK1 in lung adenocarcinoma tissues was higher than that in normal tissues, the expression in advanced cancers was higher than that in early stages, and the experimental validation revealed that among 59 cases of lung adenocarcinoma, there were 15 cases of negative expression (25.4%), 18 cases of weakly positive expression (30.5%), and 26 cases of strongly positive expression (44.1%). The different expression of DKK1 is related to methylation, prognosis and the activities of various immune cells. Functional enrichment shows that DKK1 may be involved in skin development and cell-substrate junction, and Kyoto Encyclopedia of Genes and Genomes (KEGG) analysis shows that DKK1 is related to ABC transporters. Bioinformatics analysis and clinical case specimens showed that high DKK1 expression was associated with poorer prognosis in patients with lung adenocarcinoma.

**Conclusion:**

High expression of DKK1 in lung adenocarcinoma is associated with poor prognosis. DKK1 is closely associated with tumor immune cell infiltration and pathways. DKK1 can be considered as a potential prognostic marker and a novel target for immunotherapy of lung adenocarcinoma.

肺腺癌（lung adenocarcinoma, LUAD）是肺癌的主要类型，属于非小细胞肺癌（non-small cell lung cancer, NSCLC），在所有NSCLC亚型中，LUAD有着最大的异质性和侵袭性，通常从黏膜腺发展而来，并且与多种基因的突变密切相关，有着高的肿瘤突变负荷^[1]^。LUAD分为非典型腺瘤样增生（atypical adenomatous hyperplasia, AAH）、原位腺癌（adenocarcinoma in situ, AIS）、微浸润腺癌（minimally invasive adenocarcinoma, MIA）和浸润性腺癌（invasive adenocarcinoma, IAC）四大类。LUAD具有恶性程度低、发展缓慢但容易发生血道和淋巴道转移的特点，吸烟、家族遗传史和职业暴露等是主要的危险因素，加上多数患者在就诊时已是中晚期，容易失去最佳治疗时机，因此寻找新的治疗靶点对改善预后有重要意义。近年来，随着早诊早治项目的开展，我国正在逐步建立起肺癌早筛的工作网络，发掘LUAD中差异表达的基因，可辅助高风险人群的早期筛查，并有望成为可靠的治疗和预后评估的新靶点。血清分泌型蛋白1（Dikkopf 1, DKK1）是Dikkopf家族的一员，其家族成员还包括DKK2、DKK3和DKK4。DKK1是一种由266个氨基酸组成的蛋白质，是Wnt信号通路的负向调控因子，其C末端结构域被确定为是完成Wnt通路抑制所必要的结构，同时DKK1也参与c-Jun NH2-末端激酶（JNK）和DKK1/CKAP4信号通路。在膀胱癌中DKK1的表达升高与淋巴结转移之间存在正相关；在骨骼相关疾病中高DKK1表达会损害成骨细胞活性并导致骨质流失；在心血管疾病中DKK1的表达水平已被证明与冠状动脉粥样硬化程度呈负相关，DKK1高表达也是NSCLC、食管鳞状细胞癌、胃癌、胰腺癌、前列腺癌、胆管癌、喉鳞状细胞癌和肝细胞癌等多种癌症诊断和预后的生物标志物^[2]^。同时DKK1水平升高与血管浸润和转移、淋巴侵袭以及血管内皮生长因子-C表达有关，提示DKK1可能参与癌细胞转移并在癌症的发生发展中发挥作用^[3]^。有研究^[4,5]^报道在NSCLC中，DKK1表达水平不仅高于健康对照组且表达与肿瘤直径、淋巴结转移、病理分化以及临床分期等因素相关，推测DKK1可能是LUAD潜在的生物标志物。

本研究通过生物信息学方法分析DKK1在LUAD发生发展中的作用及预后意义，同时加以实验进行验证，以期为LUAD患者的预后评估和免疫治疗提供新思路。

## 1 资料与方法

### 1.1 DKK1的表达差异分析

从基因型组织表达（genotype-tissue expression, GTEx）和癌症基因组图谱（The Cancer Genome Atlas, TCGA）数据库获取LUAD中DKK1 mRNA表达矩阵，使用Wilcoxon检验分析DKK1在LUAD组织与正常对照组织中的表达。基于DKK1的中值表达，将样本分为高和低DKK1表达组，使用“deseq 2”R软件包以logfc>1.5和P<0.05作为阈值参数，ggplot2[3.3.6]R包对火山图和热图进行可视化。

### 1.2 临床病理特征相关性分析

从TCGA数据库获取LUAD的转录组测序数据以及临床数据，对DKK1的表达与临床-患者的病理参数关系进行研究，箱型图从UALCAN数据库下载。

### 1.3 免疫细胞浸润分析

使用TISIDB数据库检索DKK1表达与LUAD免疫细胞浸润水平的联系。

### 1.4 预后分析

应用Kaplan-Meier Plotter数据库，使用Log-rank检验，通过“Lung cancer mRNA”模块探索DKK1与LUAD患者的总生存期（overall survival, OS）、首次进展生存期（first progression survival, FP）和进展后生存期（post-progression survival, PPS）之间的关系。

### 1.5 甲基化水平分析

应用Methsurv数据库分析LUAD TCGA数据集中DKK1的DNA甲基化状态，此外还在LUAD样本中评估了DKK1的CpG甲基化状态的预后价值。

### 1.6 功能富集分析

从LinkedOmics数据库LUAD数据集中获取DKK1的共表达基因，clusterProfiler[4.4.4]包进行富集分析，ggplot2包进行可视化，通过Fisher精确检验进行基因本体（gene ontology, GO）和京都基因与基因组百科全书（Kyoto Encyclopedia of Genes and Genomes, KEGG）富集显著性分析。

### 1.7 免疫组织化学染色（immunohistochemistry, IHC）

经过新疆医科大学附属肿瘤医院伦理委员会批准（批准号：K-2022016），收集2016至2017年医院手术的59例石蜡包埋的LUAD样本，其中男性27例，女性32例；年龄42-73岁，中位年龄57岁；临床分期I期12例，II期19例，III期24例，IV期4例。从医院信息中心获得其随访信息。应用Kaplan-Meier曲线经Log-rank分析后比较高DKK1表达组和低DKK1表达组生存率是否存在统计学差异。链霉菌抗生物素蛋白-过氧化物酶连结法（streptavidin-perosidase, SP）检测石蜡包埋的LUAD病理样本切片，经过二甲苯脱蜡，抗原修复，二氨基联苯胺（3,3’-diaminobenzidine, DAB）显色等步骤进行免疫组化染色，其中DKK1单克隆抗体购自Abcam公司，配套免疫组化通用试剂购自北京中杉金桥公司。DKK1染色评价标准：（1）阴性（-）：肿瘤细胞胞浆未见褐色颗粒；（2）弱阳性（+）：肿瘤细胞胞浆中见染色较浅的褐色颗粒；（3）强阳性（++）：超过50%的肿瘤细胞胞浆中见棕褐色颗粒。所有病例免疫组化结果由两位经验丰富的高级职称病理医师进行综合评估。

### 1.8 统计学方法

采用各个数据库默认参数进行统计学分析，组间比较采用Wilcoxon检验或χ^2^检验，相关性分析采用Spearman相关系数，*P*<0.05为差异有统计学意义。

## 2 结果

### 2.1 DKK1的表达差异

我们获取目标基因在LUAD的表达情况，如图1A、图1B所示。我们发现在TCGA联合GTEx数据库的862个样本中，DKK1 mRNA在LUAD组织中的表达高于健康组织（P<0.05）。此外，共鉴定了539个差异表达基因，其中270个表达上调，269个表达下调（图1C），与低表达组相比热图显示了前10个重要的差异基因（图1D），其中BRDT、MAGEA10、MAGEA4和CT83与肺癌的关系曾有报道。

**图1:**
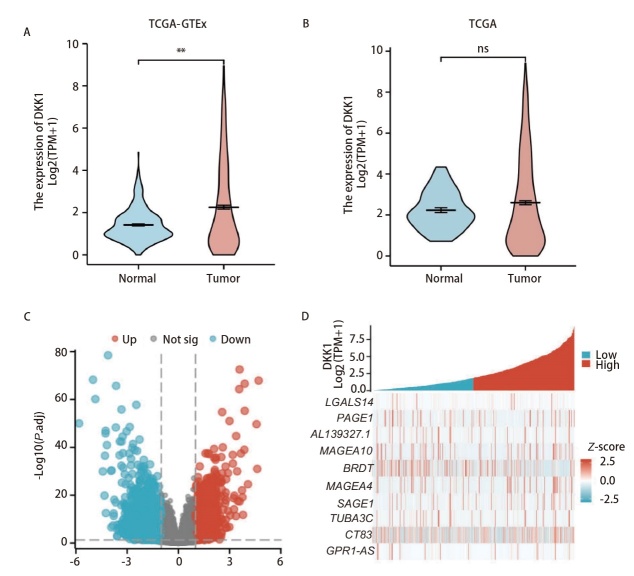
DKK1的表达图谱。A：TCGA联合GTEx数据库中LUAD样本的DKK1表达情况；B：TCGA数据库中LUAD样本的DKK1表达情况；C：DKK1高表达组和低表达组的差异基因表达热图；D：前10个重要的差异基因。

### 2.2 DKK1表达的临床病理特征

与正常对照组相比，LUAD患者具有更高程度的DKK1 mRNA表达，相较于早期癌症阶段，DKK1在晚期癌症中的表达较高（图2A，图2B），表明了DKK1在肿瘤发展和迁移中的潜在作用。图2C和图2D显示的则是DKK1的表达与患者性别以及TP53基因突变状况的关系。表1则表明DKK1表达与患者的诊断年龄相关，而与其他临床病理参数无关。

**图2:**
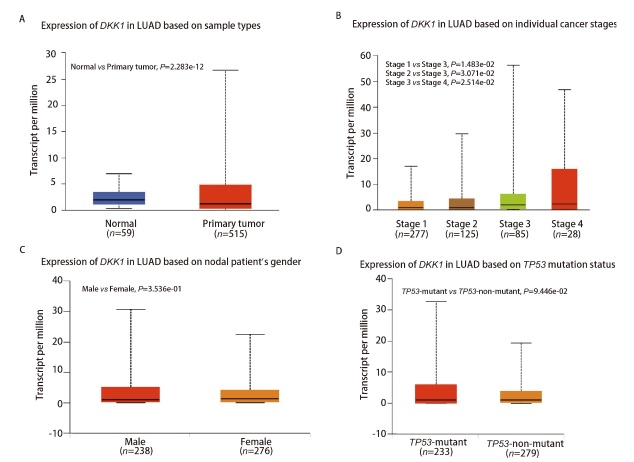
UALCAN数据库中*DKK1* mRNA表达的临床病理特征。A：样本类型（健康/原发肿瘤）；B：肿瘤分期（1、2、3和4期）；C：患者性别（男性和女性）；D：TP53基因突变状态（突变和未突变）。

**表1:** DKK1高表达组和低表达组的LUAD患者的临床病理特征

Characteristics	Low expression of DKK1 (*n*=269), *n* (%)	High expression of DKK1 (*n*=270), *n* (%)	Statistic data	*P*
Pathologic T stage			0.668	0.716
T1	90 (16.8)	86 (16.0)		
T2	148 (27.6)	144 (26.9)		
T3-T4	31 (5.8)	37 (6.9)		
Pathologic N stage			2.394	0.302
N0	176 (33.7)	174 (33.3)		
N1	52 (9.9)	45 (8.6)		
N2-N3	32 (6.1)	44 (8.4)		
Pathologic M stage			0.453	0.501
M0	186 (47.7)	179 (45.9)		
M1	11 (2.8)	14 (3.6)		
Age (yr)			11.116	0.001
≤65	148 (28.5)	109 (21.0)		
>65	113 (21.7)	150 (28.8)		
Smoker			0.212	0.645
No	37 (7.0)	40 (7.6)		
Yes	228 (43.4)	220 (41.9)		

*Data acquisition: Download and sort out RNAseq data of STAR process of TCGA-LUAD project from TCGA database and extract TPM format data and clinical data.

Data filtering strategy: remove normal+remove no clinical information.

Missing value processing: Variable missing is not uniformly processed (Pathologic T stage: 3 cases; Pathologic N stage: 16 cases; Pathologic M stage: 149 cases; Age: 19 cases; Smoker: 14 cases).

Data processing method: log2(value+1).

### 2.3 DKK1的表达与LUAD免疫细胞浸润的关系

本研究发现DKK1表达与多种免疫细胞的活动相关，其中与嗜中性粒细胞（r=0.216, P<0.001）、伽马三角洲T细胞（T gamma delta, Tgd/γδ T）（r=0.155, P<0.001）、活化树突细胞（activated dendritic cell, aDC）（r=0.149, P<0.001）以及Th1细胞（r=0.132, P=0.002）表达水平呈正相关，与滤泡辅助性T细胞（follicular helper T cell, TFH）和Th17细胞呈负相关（图3A-图3E）。上述结果促使我们研究DKK1表达水平和免疫细胞浸润之间的关系，当DKK1表达分为高表达组和低表达组时，我们发现浸润性免疫细胞水平存在显著差异，包括aDC、嗜中性粒细胞、Tgd细胞、Th1细胞、Th2细胞、DC和自然杀伤（natural killer, NK）细胞，而肥大细胞没有显著差异（图4A-图4H）。

**图3:**
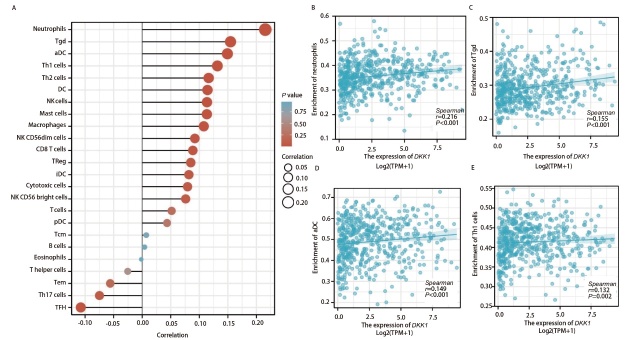
LUAD中*DKK1*表达与免疫细胞浸润的关系。*DKK1*在24种免疫细胞中表达水平的棒棒糖图（A），*DKK1*表达与中性粒细胞（B）、Tgd细胞（C）、aDC细胞（D）和Th1细胞（E）之间的相关性。

**图4:**
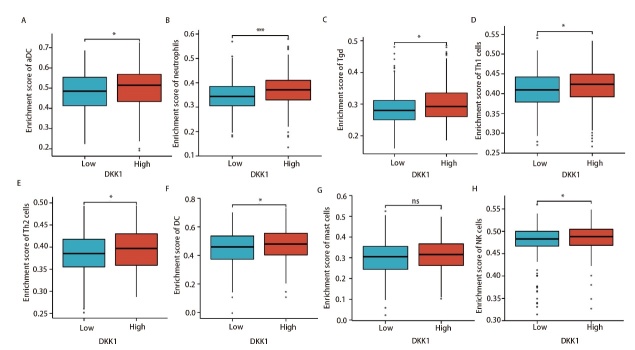
DKK1高表达组和低表达组之间aDC（A）、嗜中性粒细胞（B）、Tgd（C）、Th1（D）、Th2（E）、DC（F）、肥大细胞（G）和NK细胞（H）浸润水平的比较。

### 2.4 DKK1对LUAD患者预后的影响

预后分析显示，在204602-at（OS: HR=1.38, P=3.6e-07; FP: HR=1.63, P=2.8e-07; PPS: HR=1.26, P=0.028）的LUAD队列中，DKK1高表达组和低表达组的OS、和FP和PPS差异均存在差异（图5），DKK1表达增加与患者较差的预后有关。提示DKK1的表达水平可能对LUAD患者生存预后产生一定影响。

**图5:**
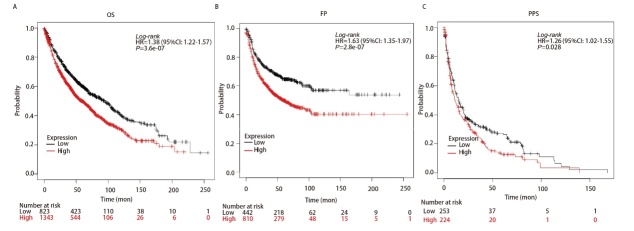
Kalpan-Meier生存曲线比较肺腺癌中DKK1的表达：肺腺癌（204602-at）队列中OS（A）、FP（B）和PPS（C）的生存曲线

### 2.5 LUAD患者1、3和5年OS的预后列线图

构建以T分期、肿瘤状态、病理分期和DKK1表达水平为参数的列线图模型（图6A），列线图模型的一致性指数（concordance index, C-index）为0.709（95%CI: 0.686-0.733）。通过受试者操作特征（receiver operating characteristic, ROC）曲线分析评估了列线图的预测能力。在TCGA-LUAD队列中，1、3和5年OS的列线图曲线下面积分别为0.735、0.643和0.599（图6B），提示该模型具有良好的预测价值。此外，通过校准曲线来评估列线图的一致性，结果显示3、5年OS率校正曲线与理想参考线接近（图6C），表示预测值和实际生存率之间具有良好的一致性。

**图6:**
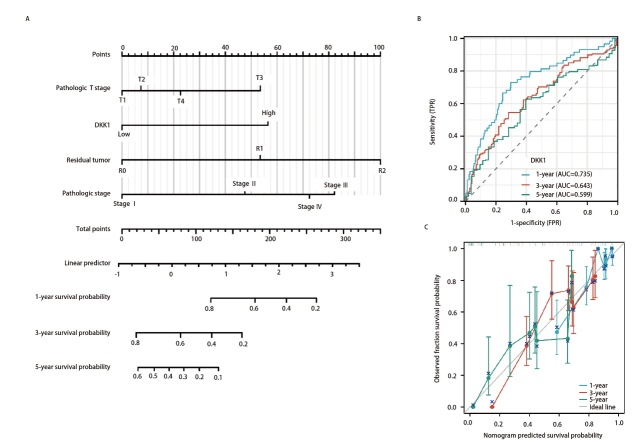
肺腺癌患者1、3和5年OS的预后列线图。A：列线图模型；B：用于预测TCGA-LUAD队列中1、3和5年OS列线图的ROC曲线；C：用于预测TCGA-LUAD队列中1、3和5年OS的列线图校准曲线。

### 2.6 DKK1的甲基化状态与LUAD患者的预后有关

与正常样本相比，LUAD中DKK1的DNA甲基化水平显著降低，但在不同癌症分期中观察到DKK1甲基化水平差异没有统计学意义（图7A-图7B）。MethSur分析了DKK1基因中DNA甲基化水平和CpG岛的预后值。结果显示了18个甲基化的CpG岛，其中cg20364839显示DNA甲基化程度最高（图7C）。此外，9个CpG岛的甲基化水平，即cg01160882、cg076844796、cg08812555、cg09445939、cg09465786、cg1193116、cg18956393、cg27411220和cg27591349与预后有关（P<0.05）（表2），表明DKK1的甲基化水平有望成为LUAD预后生物标志物。

**图7:**
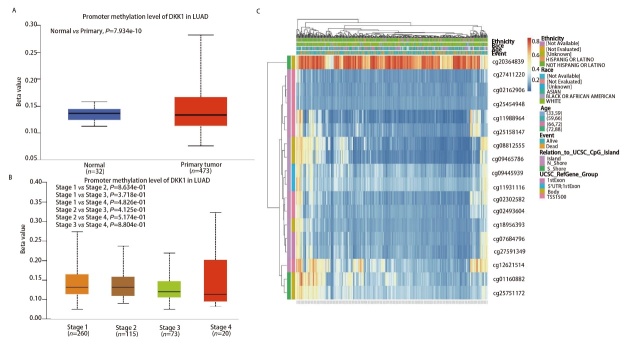
DKK1的DNA甲基化水平及其在LUAD中的预后价值。A：正常组织和肺腺癌组织中DKK1启动子甲基化水平；B：不同肿瘤阶段肺腺癌组织中DKK1启动子甲基化水平；C：Methsurv数据库DKK1基因CpG位点DNA甲基化的热图。

**表2:** DKK1基因CpG位点甲基化水平对LUAD患者预后的影响

CpG island	HR	P
BodyS_Shorecg01160882	0.618	0.005
TSS1500N_Shorecg02162906	1.241	0.178
TSS1500N_Shorecg02302582	0.810	0.189
TSS1500N_Shorecg02493604	0.783	0.175
1stExonIslandcg07684796	0.662	0.016
BodyIslandcg08812555	0.637	0.005
5'UTR; 1stExonIslandcg09445939	0.581	0.001
BodyIslandcg09465786	0.681	0.017
5'UTR; 1stExonIslandcg11931116	0.703	0.043
TSS1500N_Shorecg11988964	0.865	0.369
TSS1500N_Shorecg12621514	0.799	0.204
BodyIslandcg18956393	0.709	0.033
BodyS_Shorecg20364839	0.911	0.568
TSS1500N_Shorecg25158147	1.157	0.409
TSS1500N_Shorecg25454948	1.250	0.165
BodyS_Shorecg25751172	0.731	0.072
TSS1500N_Shorecg27411220	0.535	<0.001
1stExonIslandcg27591349	0.547	<0.001

### 2.7 DKK1在LUAD中的功能富集分析

我们利用LinkedOmics数据库探索了DKK1共表达网络，验证了DKK1在LUAD中的潜在功能及作用。在LUAD中，2398个基因（深红点）与DKK1呈显著正相关，2399个基因（深绿点）呈负相关（FDR<0.01）（图8A），使用热图显示与DKK1呈正负相关的前50个基因（图8B，图8C），其中CREG2、GJB3、TNS4、PLCD3与DKK1表达相关性最强（r=0.478、0.467、0.461、0.459和P=1.05e-30、3.17e-29、1.87e-28、3.75e-28）。我们使用R软件包对DKK1相关基因进行GO和KEGG富集分析。GO功能注释表明DKK1共表达主要参与皮肤发育、细胞-基质连接、钙依赖性磷脂结合；KEGG通路分析表明DKK1共表达主要与ABC转运蛋白相关（图8D）。

**图8:**
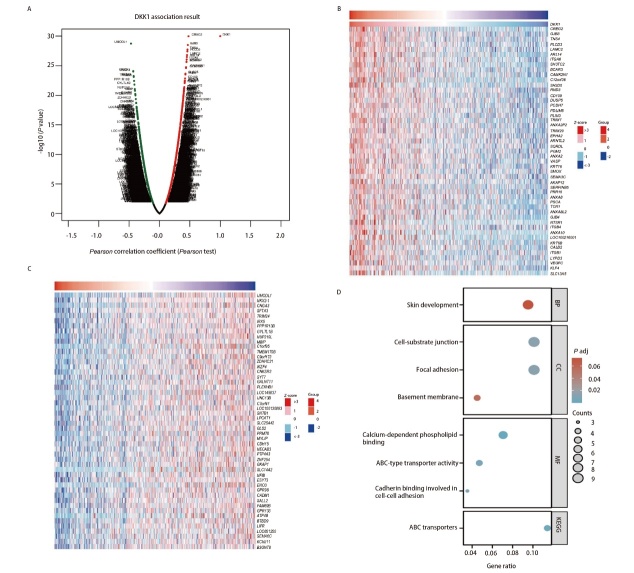
DKK1在LUAD中的功能富集分析。A：LinkedOmics数据库DKK1共表达网络图；B：前50个正相关基因的热图；C：前50个负相关基因的热图；D：GO和KEGG富集分析。

### 2.8 IHC对DKK1在LUAD中高表达的验证

我们针对DKK1在LUAD中的表达及其预后价值来证实上述发现，选取来自新疆医科大学附属肿瘤医院的59例石蜡包埋的LUAD样本进行IHC研究，结果发现，59例LUAD中阴性表达15例（25.4%，图9A），弱阳性表达18例（30.5%），强阳性表达26例（44.1%，图9B）。对59例患者进行随访，其中4例失访，DKK1低表达组包括阴性和弱阳性，共33例；高表达组为DKK1强阳性表达，共26例。两组生存率存在统计学差异（χ^2^=3.998, P=0.042），DKK1低表达组生存率更高（图9C）。

**图9:**
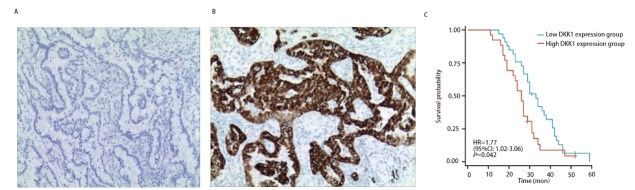
DKK1特性的初步实验验证。A：DKK1表达为阴性的免疫组化图（×100）；B：DKK1表达为阳性的免疫组化图（×100）；C：59例LUAD患者不同DKK1表达量分组预后情况。

## 3 讨论

肺癌的诊治一直以来都是临床的研究重点，有报告^[6]^指出肺癌是全球癌症致死的常见原因，占癌症致死总数的18%，近年来，我国肺癌的5年生存率提高了3.6%，但仍有75%的患者处于肺癌晚期，因此需要一个新的指标来精准预测预后^[7]^。DKK1于1998年在非洲蟾蜍胚胎细胞中发现，由两个富含半胱氨酸的保守结构域和一个50-55个氨基酸组成的连接区域构成，主要通过与LRP5/6结合并阻断Wnt相互作用来抑制Wnt信号传导，人类DKK1基因位于染色体10q11.2.26上^[8⇓-10]^。DKK1影响肿瘤发生机制的研究^[11⇓-13]^显示，DKK1通过调控Wnt信号通路，从而对肿瘤细胞生长、增殖、侵袭转移产生影响，对血管生成、肿瘤免疫微环境、化疗耐药性的影响也有报道。有关DKK1在NSCLC中的生物学效应有研究^[14]^发现DKK1的过表达促进了人NSCLC细胞系95C的迁移和侵袭；Zhang等^[15]^发现DKK1可以诱导上皮间质转化，在促进细胞系迁移和侵袭性生长方面发挥作用；姚伶俐等^[16]^通过建立H460-DKK1裸鼠移植瘤模型证实了DKK1高表达促进人NSCLC中线性程序性死亡（linearly patterned programmed cell necrosis, LPPCN）和血管生成拟态（vasculogenic mimicry, VM）形成。近年来，生物信息学研究成为热点，但DKK1在LUAD中的作用研究较少，故本研究旨在通过生物信息学方法探索DKK1在LUAD中的表达和预后情况以及其在肿瘤免疫微环境中的作用和潜在机制。

有关DKK1在癌症中的表达，一方面认为DKK1在胆管癌^[17]^、食管癌^[18]^、头颈癌^[19]^、肝癌^[20]^、NSCLC^[4]^、胃癌^[21]^和乳腺癌^[22]^中表达上调，另一方面认为DKK1在卵巢癌^[23]^和结直肠癌^[24]^中表达下调。我们在TCGA联合GTEx数据库中发现DKK1在LUAD组织中较正常肺组织表达明显上调，同时通过来自新疆医科大学附属肿瘤医院的LUAD样本验证发现，59例LUAD中阴性表达占25.4%，弱阳性表达占30.5%，强阳性表达占44.1%，实验验证结果与从数据库得出的结果一致，证明DKK1在LUAD中的高表达。研究DKK1与LUAD患者病理特征之间的关系时我们发现，DKK1在晚期癌症中表达高于早期阶段，表明潜在的DKK1在肿瘤发展和迁移中的作用。王中秋等^[25]^研究发现DKK1的表达与NSCLC患者的病理分级、淋巴结转移、肿瘤原发灶-淋巴结-转移（tumor-node-metastasis, TNM）分期、远处转移和血管浸润有关，而本研究发现DKK1表达与患者的诊断年龄相关，与肿瘤的TNM分期、患者性别以及TP53基因突变等无关。越来越多的研究^[26]^证明，免疫细胞浸润是肿瘤进展和免疫治疗的关键因素，也有研究^[11]^表明，DKK1与多种癌症的免疫细胞浸润密切相关，本研究发现DKK1与aDC、嗜中性粒细胞和Tgd等多个免疫细胞浸润有关，推断DKK1可能通过影响免疫细胞的功能、调节免疫细胞活性或Wnt信号通路来影响肿瘤的发生发展。少有研究说明DKK1甲基化在LUAD中的可能性，我们观察到与正常组织相比LUAD中的DKK1启动子甲基化水平显著降低，在研究DKK1甲基化水平与LUAD患者预后的关系中，我们发现有9个CpG位点的甲基化水平与预后相关，我们推测DKK1可能通过甲基化促进LUAD的发生。生存分析结果显示DKK1高表达组的PPS明显缩短，提示DKK1高表达与患者预后不良有关，通过对55例患者的随访，我们发现DKK1低表达组有更高的生存率，这与我们得出的结果一致。预后列线图模型显示在TCGA-LUAD队列中，1、3和5年OS的列线图曲线下面积均大于0.5，且3和5年预测值和实际生存率之间具有良好的一致性。与BRDT、MAGEA10、MAGEA4和CT83等差异基因进行联系后我们发现，CT83表达与LUAD患者的预后相关^[27]^；而BRDT的异常激活首先在肺癌中检测到^[28]^，MAGEA10、MAGEA4也在肺癌中表达^[29,30]^。GO分析发现，DKK1及其共表达基因主要参与皮肤发育、细胞-基质连接、钙依赖性磷脂结合。KEGG通路表明，DKK1共表达主要与ABC转运蛋白相关，有研究^[31]^报道在肺癌中ABC转运蛋白表达与不同的肿瘤分期和人群相关。至于DKK1与ABC转运蛋白是如何影响LUAD的发生还需要进一步进行探究。

综上所述，本研究主要通过生物信息学方法分析DKK1在LUAD中的表达，发现DKK1在LUAD免疫治疗和预后预测方面的潜在价值，并初步探讨了DKK1在LUAD中可能参与的信号通路。同时我们的研究也存在一定的局限性，实验验证采用的是回顾性研究，可能存在一定的选择偏倚，再加上标本量的原因，需要后续加大样本量来进行验证。

**Competing interests**

The authors declare that they have no competing interests.
